# Characterization of Sugar Contents and Sucrose Metabolizing Enzymes in Developing Leaves of *Hevea brasiliensis*

**DOI:** 10.3389/fpls.2018.00058

**Published:** 2018-02-01

**Authors:** Jinheng Zhu, Jiyan Qi, Yongjun Fang, Xiaohu Xiao, Jiuhui Li, Jixian Lan, Chaorong Tang

**Affiliations:** ^1^Rubber Research Institute, Chinese Academy of Tropical Agricultural Sciences, Danzhou, China; ^2^College of Tropical Agriculture and Forestry, Hainan University, Haikou, China; ^3^Institute of Tropical Bioscience and Biotechnology, Chinese Academy of Tropical Agricultural Sciences, Haikou, China

**Keywords:** rubber tree, leaf development, sucrose metabolism, sugar content, enzyme activity, gene expression

## Abstract

Sucrose-metabolizing enzymes in plant leaves have hitherto been investigated mainly in temperate plants, and rarely conducted in tandem with gene expression and sugar analysis. Here, we investigated the sugar content, gene expression, and the activity of sucrose-metabolizing enzymes in the leaves of *Hevea brasiliensis*, a tropical tree widely cultivated for natural rubber. Sucrose, fructose and glucose were the major sugars detected in *Hevea* leaves at four developmental stages (I to IV), with starch and quebrachitol as minor saccharides. Fructose and glucose contents increased until stage III, but decreased strongly at stage IV (mature leaves). On the other hand, sucrose increased continuously throughout leaf development. Activities of all sucrose-cleaving enzymes decreased markedly at maturation, consistent with transcript decline for most of their encoding genes. Activity of sucrose phosphate synthase (SPS) was low in spite of its high transcript levels at maturation. Hence, the high sucrose content in mature leaves was not due to increased sucrose-synthesizing activity, but more to the decline in sucrose cleavage. Gene expression and activities of sucrose-metabolizing enzymes in *Hevea* leaves showed striking differences compared with other plants. Unlike in most other species where vacuolar invertase predominates in sucrose cleavage in developing leaves, cytoplasmic invertase and sucrose synthase (cleavage direction) also featured prominently in *Hevea*. Whereas SPS is normally responsible for sucrose synthesis in plant leaves, sucrose synthase (synthesis direction) was comparable or higher than that of SPS in *Hevea* leaves. Mature *Hevea* leaves had an unusually high sucrose:starch ratio of about 11, the highest reported to date in plants.

## Introduction

Mature leaves are the main photosynthetic organs that fix atmospheric carbon to produce sucrose to feed non-photosynthetic tissues via phloem-mediated long distance transport in most plants (Lambers et al., 1998). Newly emerging leaves, being sink tissues, are net importers of assimilates. They pass through developmental stages of varying durations according to the species until they assume the role of net exporters of assimilates. Such development involves structural and physiological changes, in which the participation of enzymes in carbohydrate metabolism is noteworthy (Foyer and Paul, 2001). Normal leaf formation and maturation is critical not only to growth, development and defense responses of plants themselves but to productivity in agriculture.

Sucrose metabolism plays fundamental roles in plant leaf development. In the past three decades, rapid progress has been achieved in understanding the dynamics of sucrose-metabolizing enzymes during leaf development (Turgeon, 1989; Foyer and Paul, 2001; Ruan, 2014). Sucrose-metabolizing enzymes are functionally divided into cleavage and synthesis groups. The cleavage group includes invertase and sucrose synthase (Sus). The former hydrolyzes sucrose irreversibly into two reducing hexose sugars (glucose and fructose), whereas the latter cleaves sucrose reversibly in the presence of UDP into UDP-glucose (UDPG) and fructose. According to subcellular locations and optimum pH, invertase is classified into cell-wall, vacuolar, and cytoplasmic isoforms, designated CWI, VIN and NIN, respectively (Tymowska-Lalanne and Kreis, 1998). CWI and VIN are characterized by an acidic pH optimum (3.5–5.5), whereas NIN by an alkaline or neutral pH optimum (6.8–8.0) (Roitsch and González, 2004). In nearly all plant species explored to date (Schaffer et al., 1987; Singh and Luthra, 1988; Huber, 1989; Turgeon, 1989; Ricaud et al., 1994; Alaoui-Sossé et al., 1996; Ruan, 2014), VIN is the major sucrose-cleaving enzyme during leaf development whereas NIN and Sus appear much less important. Sucrose synthesis is catalyzed by two distinct enzymes in higher plants: sucrose phosphate synthase (SPS) and Sus. Using UDPG and Fructose-6-P as substrates, SPS synthesizes sucrose-6-P that is then dephosphorylated into sucrose by sucrose phosphate phosphatase (SPP) (Huber and Huber, 1996). Sus synthesizes sucrose exactly in the reverse reaction as it cleaves sucrose. Generally, SPS rather than Sus is regarded as the main enzyme responsible for sucrose synthesis in leaves (Winter and Huber, 2000; Ren and Zhang, 2013; Wang et al., 2013). In developing leaves, sucrose-cleaving enzymes, especially VIN, are very active; they contribute to rapid leaf growth by providing high amounts of reducing sugars as substance to form cell carbon skeletons and fuel various energy-consuming mechanisms (Pantin et al., 2012). In mature leaves, however, sucrose-cleaving enzymes usually maintain much lower activities compared to young leaves, whereas sucrose-synthesizing enzymes, especially SPS, exhibit high activities. In recent years, transgenic studies have been conducted to explore the effects of sucrose metabolizing enzymes on leaf development. In transgenic cotton overexpressing a potato Sus gene, expansion of young leaves is accelerated with significantly increased fructose concentrations and a slight decline for sucrose (Xu et al., 2012). Similar results are observed in transgenic tobacco overexpressing a loquat VIN (Wang et al., 2015). Existing knowledge on sucrose-metabolizing enzymes during leaf development has mainly been drawn from studies on temperate species (Huber, 1989; Foyer and Paul, 2001; Roitsch and González, 2004; Ruan, 2014). It is hence worthwhile considering whether the general understanding thus acquired could be extended to the entire plant kingdom.

The rubber tree (*Hevea brasiliensis*; *Hevea* hereafter) is an important tropical species, being the sole commercial source of natural rubber (*cis*-1,4-polyisoprene) (van Beilen and Poirier, 2007). *Hevea* uses sucrose as the precursor molecule for rubber biosynthesis and latex regeneration in rubber-producing laticifers (Tupy, 1989). Physiological and molecular regulation of sucrose metabolism has been intensively studied in relation to rubber production in the laticifers of the *Hevea* trunk, which is the site tapped for latex (Tupy, 1989; Dusotoit-Coucaud et al., 2010; Tang et al., 2010; Liu et al., 2015, 2016). By comparison, very few studies are available with regard to sucrose metabolism in *Hevea* leaves, although the co-existence of VIN and NIN isoforms has been shown in both growing and developed *Hevea* leaves (de Oliveira et al., 2006). *Hevea* leaf development has been investigated mainly with respect to leaf diseases (Lieberei et al., 1989; Voss, 2001; Kursar and Coley, 2003; Lieberei, 2007), especially South American leaf blight (SALB), a fungal disease that has hampered *Hevea* planting in South and Central America. Cyanogenic metabolism, lignin and anthocyanin biosynthesis have been shown to correlate closely with the susceptibility of *Hevea* leaves during development (Lieberei, 2007; Fang et al., 2016).

This study aimed to look at the correlation between sucrose metabolizing enzymes and transcript levels during four different developmental stages in *Hevea* leaves in an attempt to fill the gap outside temperate species.

## Materials and Methods

### Plant Materials

*Hevea* leaves at four progressive developmental stages (Fang et al., 2016), *i.e.*, bronze (I), color-change (II), pale green (III), and bright green (mature; IV) were sampled at 10 a.m. from untapped 4-year-old tissue-cultured plants of the cultivar Reyan7-33-97 that were cultivated in the experimental plantation of the Rubber Research Institute, Chinese Academy of Tropical Agricultural Sciences (Danzhou, Hainan, China). Three individual trees with similar girth and growth vigor were chosen, each of which was used as a separate biological repeat.

### Enzyme Extraction

Leaf soluble proteins were extracted according to the method of Walker and Huber (1989) with slight modifications. Fresh leaves (0.2 g) were ground using a mortar and pestle in 2 mL ice cold extraction buffer containing 50 mM HEPES-NaOH (pH 7.5), 1 mM EDTA, 5 mM Mg^2+^, 10 mM β-mercaptoethanol, 0.05% (v/v) Triton X-100 and 1% PVP40 (w/v). The homogenate was centrifuged at 20,000 *g* for 10 min. The supernatant was desalted and made free of soluble sugars on a Sephadex G-25 column equilibrated with extraction buffer minus the Triton X-100 and PVP40 prior to four enzyme assays, viz. VIN, NIN, Sus, and SPS.

To extract CWI, the centrifugation pellet was suspended in 100 μL of 1 M NaCl and 400 μL of extraction buffer, and kept at 4°C overnight. The suspension was centrifuged at 20,000 *g* for 10 min with the supernatant used for the CWI assay.

Protein concentrations of enzyme extract were determined according to the method of Bradford (1976).

### Enzyme Assays

Invertase activities were determined as described previously with modifications (Tupý, 1969; Miron and Schaffer, 1991). Assays were tested in a reaction mixture of 100 μL containing 30 μL enzyme extract and 100 mM sucrose in the reaction buffer (50 mM HEPES, 5 mM Mg^2+^ and 1 mM EDTA). The pHs of the reaction buffers were adjusted to 7.5, 4.5, and 3.5, respectively, for the three invertases: NIN, VIN and CWI. The mixture was chilled on ice immediately after incubation at 37°C for 1 h and then adjusted to 1mL by adding 900 μL of 3, 5-dinitrosalicylic acid reagent (DNS), followed by 5 min of incubation in a boiling water bath. The blank control contained 30 μL of boiled enzyme extract. Contents of reducing sugars produced in the reaction mixture were measured using dinitrosalicylic acid reagent in conformity with Miller (1959). Enzyme activity was expressed in units per mg of protein, where one unit (U) was defined as 1 μmol of sucrose cleaved per hour under the experiment conditions.

The activity of SPS was determined by measuring the sucrose (plus sucrose-6-P) formed in the assay mixture (100 μL) that contained 30 μL enzyme extract, 5 mM UDPG, and 40 mM fructose-6-P in the reaction buffer (50 mM HEPES, 5 mM Mg^2+^ and 1 mM EDTA, pH 6.5) (Huber, 1981). After incubation at 37°C for 1 h, 100 μL of 1 M NaOH was added to terminate the reaction and destroy the unreacted hexose simultaneously by placing the tubes in boiling water for 10 min. The blank differed from the assay mixture by lacking UDPG, and it was kept on ice prior to the addition of NaOH. After chilling on ice, 250 μL of 0.1% (w/v) resorcinol in 95% ethanol and 750 μL of 30% HCl were added to each tube, followed by incubation at 80°C for 10 min. The tubes were then centrifuged at 20,000 *g* for 5 min, and the OD520 was measured by a Spectramax 250 microplate reader (Molecular Devices, Sunnyvale, CA, United States). Enzyme activity was expressed in units per mg of protein, where one unit (U) was defined as 1 μmol of sucrose produced per hour.

Sus catalyzes a reversible reaction that both hydrolyzes and synthesizes sucrose. Sus activity in the synthesis direction was assayed as for SPS except that the pH of reaction buffer was changed to 8.5, and fructose-6-P substituted with fructose. Sus activity in the cleavage reaction was assayed as for invertase except that the pH of reaction buffer was changed to 6.5, and 5 mM UDP was supplemented. The blank control contained no UDP. Enzyme activities were expressed in units per mg of protein, where one unit (U) was defined as 1 μmol of sucrose produced or cleaved per hour.

In line with standard practices in enzymology, blanks are expected to contain substrates as well. However, leaving out the substrates UDPG (SPS, SSS) or UDP (SSC) from the blanks did not alter the OD_520_ readouts (Supplementary Table S1). Accordingly, HPLC-ELSD analysis of the UDPG and UDP substrates showed complete absence of sucrose and fructose (Supplementary Figure S1).

### Soluble Sugar Extraction

Leaves were desiccated at 105°C for 30 min and then at 80°C to constant weight. The dried leaves were ground into powder using a mortar and pestle in room temperature, and kept in a dryer prior to use. Soluble sugars were extracted according to Ma et al. (2014) with modifications. Approximately 100 mg was sonicated in 800 μL double-distilled water (ddH_2_O) for 30 min and then incubated at 80°C for 30 min with intermittent shaking for every 5 min. The mixture was centrifuged at 4°C at 20,000 *g* for 10 min. The supernatant was kept at -20°C, whereas the pellet was subjected to a second round of extraction with 200 μL of ddH_2_O. The supernatant pooled from the two rounds of extraction was dried under vacuum at 60°C, then dissolved in 80% ethanol (v/v) and kept at -20°C overnight. The suspension was centrifuged at 4°C in 20,000 *g* for 10 min. The supernatant was dried again, dissolved in 50% acetonitrile (v/v), and stored at -20°C before use.

To prepare phloem exudate, 1-year-old *Hevea* branches bearing mature leaves were excised with a sharp knife, and the cuts were cleaned with sterile water, and then subjected to the extraction of phloem exudate following the protocol of King and Zeevaart (1974). The exudates obtained were lyophilized, and dissolved in 50% acetonitrile (2 mL per branch), and stored at **-**20°C before use.

### Soluble Sugar Analysis by HPLC-ELSD

The high-performance liquid chromatography (HPLC)-evaporative light scattering detection (ELSD) method was used to determine soluble sugars in *Hevea* leaves and phloem exudate. HPLC analysis was performed on a Waters e2695 separations module (Waters, Milford, MA, United States) equipped with an Alltech 3300 ESLD detector (Alltech, Deerfield, IL, United States). Separation was achieved on a XBridge^TM^ Amide column [4.6 mm × 250 mm i.d., 3.5 μm particle size (Waters, Milford, MA, United States)]. All samples and standards were filtered through 0.45 μm Millipore filters before loading samples of 10 μL onto the machine. The HPLC-ELSD conditions were optimized following Ma et al. (2014) with a solvent ratio of 85 acetonitrile:15 water (v/v), a flow rate of 1 mL/min, the column and drift tube temperatures set at 45 and 82°C, respectively, and the nebulizer gas flow rate set at 2 L/min. Peaks were quantified using calibration standards of HPLC grade sugars, viz. glucose, fructose, sucrose, raffinose, and stachyose (Sigma–Aldrich, Shanghai, China), and sorbitol and quebrachitol (Shanghai Yuan ye Bio-Technology Co., Ltd., Shanghai, China).

### Analysis of Starch Content

Starch extraction and content assay in *Hevea* leaves were performed following the procedures of the Total Starch Assay Kit (Megazyme, Bray, Ireland).

### RNA Isolation and cDNA Synthesis

Total RNA of *Hevea* leaves was extracted using the Plant Total RNA Extraction Kit (BioTeke Corporation, Beijing, China) following the manufacturer’s instructions. RNA integrity was checked by agarose gel electrophoresis while RNA concentration and purity were examined using a NanoDrop 2000 spectrophotometer (Thermo Fisher Scientific, Wilmington, DE, United States). RNA samples were prepared DNA-free, and reverse transcribed to cDNA using the PrimeScript^TM^ First Strand cDNA Synthesis Kit (TaKaRa, Dalian, China) following the manufacturer’s protocol.

### Gene Expression Profile Based on RNA-Seq Analysis

The RNA-Seq data of *Hevea* leaves at four developmental stages (I to IV) in our previous study (Fang et al., 2016) were divided into two sets: developing (stage I to III) and mature (stage IV). The two data sets were exploited to determine the expression in FPKM (Fragments Per Kilobase of transcript per Million fragments mapped) of sucrose-metabolizing genes in developing and mature leaves by using the RSEM software (Li and Dewey, 2011).

### Quantitative Real-Time PCR Analysis

Specific PCR primers for the main sucrose-metabolizing genes (Supplementary Table S2) were designed by Primer Premier 5, and their specificity was confirmed by agarose electrophoresis and sequencing of the PCR products. Quantitative real-time PCR (qPCR) was carried out using the SYBR Premix Ex Taq II (2×; Tli RNaseH Plus; TaKaRa, Dalian, China) on a CFX96 Touch^TM^ Real-Time PCR detection system (Bio-Rad, Hercules, CA, United States). Quantification cycle values, PCR efficiency, correlation coefficients and the relative fold change of expression were determined as previously described (Long et al., 2015, 2016). The *Hevea YLS8* gene was selected as an internal control based on the results of our previous work (Li et al., 2011).

### Statistical Analysis

All statistical analyses were conducted using SPSS Statistics 22. Duncan’s test was used to compare sugar concentrations, enzyme activity, and gene expression in *Hevea* leaves across the four developmental stages. Differences were accepted as significant at *P <* 0.05. Correlation coefficients were determined between activity of sucrose-metabolizing enzymes and soluble sugar contents or gene expression in the course of *Hevea* leaf development. Correlation was accepted as significant at *P <* 0.05 or 0.01.

## Results

### Changes in Specific Leaf Weight during Leaf Development

In the process of *Hevea* leaf development, leaf colors changed from bronze (stage I) to color change (stage II), pale-green (stage III), and then to bright green (stage IV), identical to our previous report (Fang et al., 2016). Leaf size (cm^2^) increased rapidly from stages I (9.6 ± 0.6) to III (83.0 ± 10.6), and remained unchanged thereafter (**Table 1**). On the contrary, specific leaf weight (mg fresh weight per cm^2^) decreased progressively from stages I (12.5 ± 1.0) to III (9.8 ± 1.7), but resumed to a high value of 12.9 ± 1.5 in mature leaves (stage IV) (**Table 1**).

**Table 1:** Changes in leaf area and specific leaf weight during *Hevea* leaf development.

Leaf stage	Leaf area (cm^2^)	Specific leaf weight (mg/cm^2^)
I	9.6 ± 0.6	12.5 ± 1.0
II	26.5 ± 4.1	10.9 ± 2.6
III	83.0 ± 10.6	9.8 ± 1.7
IV	81.7 ± 7.8	12.9 ± 1.5

*Data are the means ± SE of three replicates.*

### Changes in Sugar Composition during Leaf Development

To determine soluble sugar composition in *Hevea* leaves and branch phloem exudates, the HPLC-ELSD system was used. Six sugars, namely glucose, fructose, sorbitol, sucrose, raffinose, and stachyose that are reported to be abundant or translocated in most plant species (Zamski and Schaffer, 1996) served as reference standards, together with quebrachitol which is the predominant non-rubber carbon compound in *Hevea* latex (Bealing, 1981). Raffinose and stachyose, easily separated from the five other sugars by the HPLC-ELSD method (Supplementary Figure S2A), were not detected in *Hevea* leaves and phloem exudate. Glucose, fructose, sucrose, sorbitol, and quebrachitol were well separated in 28 min by the optimized HPLC-ELSD method (Supplementary Figure S2B). Fructose, glucose, and sucrose turned out to be the main soluble sugars in *Hevea* leaves although their relative abundance varied with leaf development (Supplementary Figures S3A–D). Interestingly, *Hevea* leaves contained also small amounts of quebrachitol, but no sorbitol. *Hevea* phloem exudate from branches exhibited a profile of sugar composition similar to that of the leaf (Supplementary Figure S3E), except that sucrose was in far greater abundance than the other sugars, consistent with a previous assumption of sucrose being the major transported carbohydrate in *Hevea* (Tupy, 1989).

**FIGURE 1:**
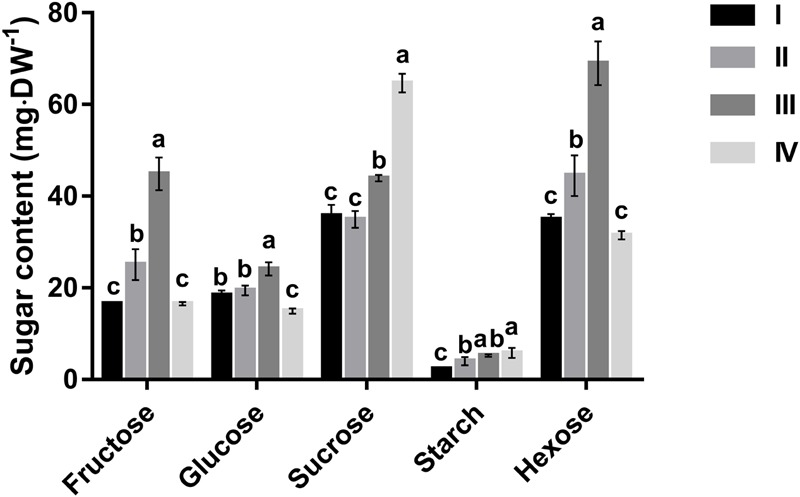
Changes in fructose, glucose, sucrose, starch and hexose concentrations of *Hevea* leaves at four developmental stages (I to IV). Values are the means ± SE of three replicates. Different letters above the bars of respective sugars indicate a significant difference (*P* < 0.05) across different developmental leaf stages.

Changes in leaf contents of glucose, fructose, sucrose, and starch during leaf development are depicted in **Figure 1**. Starch accumulated in the course of leaf development, with a 2.5-fold increase from stage I to stage IV. At all leaf stages, however, starch was much lower than the other three sugars. In mature leaves (stage IV), starch comprised only 5.7% of total sugars, whereas sucrose accounted for 63.5%. Glucose and fructose had comparable concentrations except for stage III where the content of fructose was almost double that of glucose. Concentrations of fructose and glucose increased progressively with leaf development, and their combined content was more than 1.5-fold that of sucrose, but then decreased abruptly at maturation to less than 0.5-fold that of sucrose at the same stage (**Figure 1**). The content of sucrose remained almost unchanged from stage I to II, and then increased noticeably at stage III, before peaking at maturation (stage IV).

### Activity of Sucrose-Metabolizing Enzymes during Leaf Development

**FIGURE 2:**
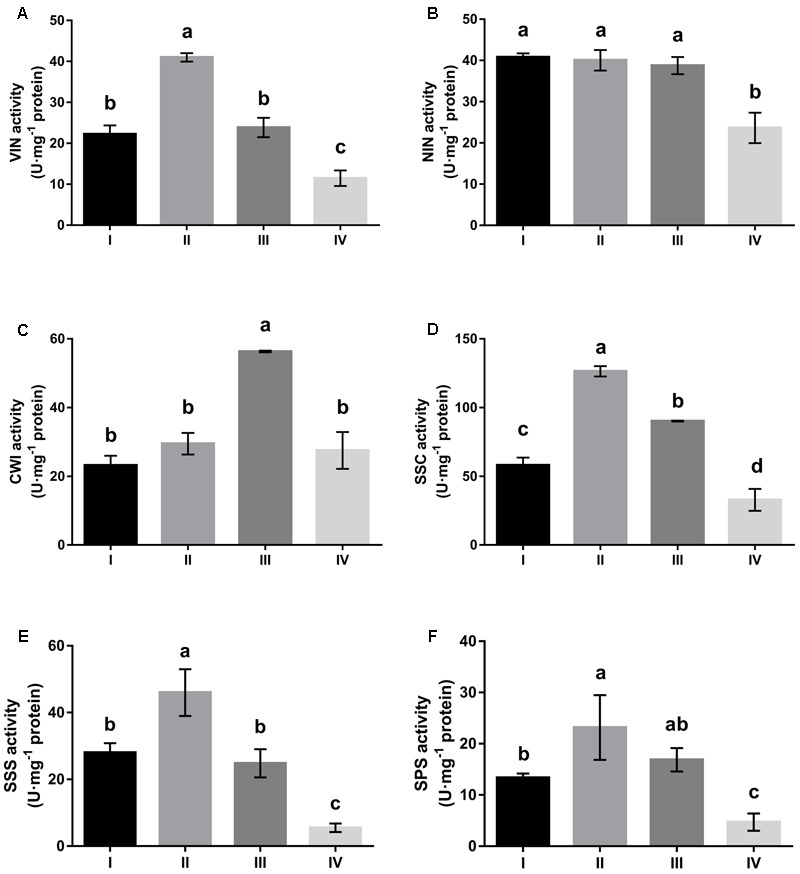
Changes in activities of sucrose-metabolizing enzymes at four stages of *Hevea* leaf development. **(A)** Vacuolar invertase (VIN); **(B)** alkaline/neutral invertase (NIN); **(C)** cell wall invertase (CWI); **(D)** sucrose synthase in the cleavage direction (SSC); **(E)** sucrose synthase in the synthesis direction (SSS); **(F)** sucrose phosphate synthase (SPS). Values are the means ± SE of three replicates. Different letters above the bars indicate a significant difference with *P* < 0.05 across different developmental leaf stages.

All sucrose-cleaving enzymes, i.e., VIN, NIN, CWI, and Sus in the cleavage direction (SSC) showed comparable activities in developing leaves (stages I to III) that were significantly higher than in mature leaves (stage IV) (**Figure 2**). Higher activity of sucrose-cleaving enzymes corresponded well to a higher hexose content in developing leaves (**Figure 1**). Examined in detail, the four sucrose-cleaving enzymes exhibited obvious variation in activity. VIN activity increased almost two-fold between stages I and II, and then decreased progressively to stage IV, reaching a level of only one fourth of its highest at stage II (**Figure 2A**). NIN activity remained constantly high throughout the three developing stages (I to III), but decreased significantly by 40% at maturation (stage IV) (**Figure 2B**). CWI and SSC activities shared a similar pattern of variation, both of which increased progressively with leaf development until stage III, and then declined abruptly at stage IV by 50% and 80%, respectively (**Figures 2C,D**). In mature leaves, NIN and CWI activities were significantly higher than those of VIN and SSC. Interestingly, sucrose-synthesizing enzymes, i.e., SPS and Sus in the synthesis direction (SSS) behaved similarly to sucrose-cleaving enzymes by displaying higher activity in developing leaves than in mature ones (**Figures 2E,F**). Correlations between activities of sucrose-metabolizing enzymes and the soluble sugar content were investigated in *Hevea* leaves during their development (**Table 2**). CWI activity correlated positively with fructose content at a significant level, whereas SSC and SSS activities showed significant positive correlation with glucose content. Interestingly, NIN activity exhibited a significant negative correlation with sucrose content, suggesting that the extent of its cleavage by NIN influenced its presence in the leaf.

**Table 2:** Correlation coefficients between activity of sucrose-metabolizing enzymes and soluble sugar content in the course of *Hevea* leaf development.

	Enzyme activity					
	NIN	CWI	VIN	SSC	SSS	SPS
Fructose	0.365	0.978^a^	0.368	0.876	0.873	0.454
Glucose	0.682	0.850	0.530	0.971^a^	0.967^a^	0.642
Sucrose	-0.978^a^	-0.009	-0.791	-0.64	-0.642	-0.849

*^a^Significant at *P* < 0.05. Correlation analysis was conducted using SPSS Statistics 22.*

### Expression of Sucrose-Metabolizing Enzymes during Leaf Development

**FIGURE 3:**
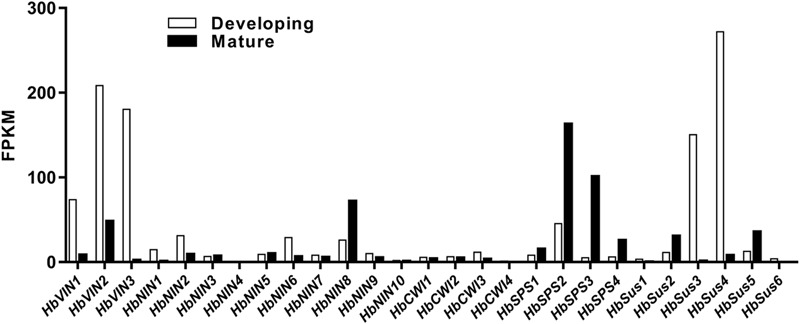
Expression of sucrose-metabolizing genes in developing and mature *Hevea* leaves determined by RNA-Seq analysis. Expression of the respective genes is represented in Fragments Per Kilobase of transcript per Million mapped reads (FPKM). Values for developing leaves are means of stages I to III.

**FIGURE 4:**
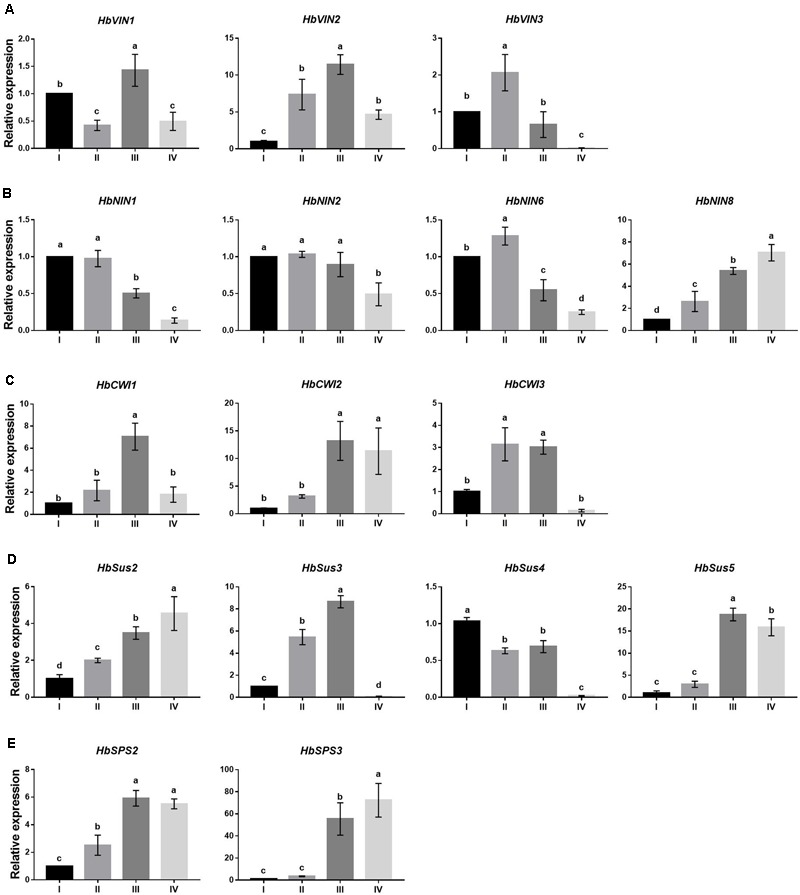
Changes in expression by qPCR of sucrose-metabolizing genes at four stages (I to IV) of *Hevea* leaf development. **(A)** Three vacuolar invertase genes, *HbVIN1, 2* and *3*; **(B)** four alkaline/neutral invertase genes, *HbNIN1, 2, 6* and *8*; **(C)** three cell-wall invertase genes, *HbCWI1, 2* and *3*; **(D)** four sucrose synthase genes, *HbSus2* to *5*; **(E)** two sucrose phosphate synthase genes, *HbSPS2* and *3*. Expression of respective genes was normalized against the level at leaf stage I. Values are means ± SE of three replicates. Different letters above the bars indicate a significant difference with *P* < 0.05 across different developmental leaf stages.

To investigate the transcriptional regulation of sucrose-metabolizing enzymes and their roles in *Hevea* leaf development, the principal encoding genes expressed in developing and mature leaves were first identified among respective gene families of sucrose-metabolizing enzymes (VIN, NIN, CWI, SPS and Sus) using the RNA-Seq data (**Figure 3**). The main members thus identified from different gene families, viz. HbVIN1 to 3, HbNIN1, 2, 6 and 8, HbCWI1 to 3, HbSus2 to 5, and HbSPS2 and 3 were explored for their expression dynamics with leaf development by qPCR analysis (**Figure 4**).

Expressions of all three *HbVIN* genes in *Hevea* leaves decreased significantly between stages III and IV (**Figure 4A**), corresponding well to an abrupt depression in VIN activity at stage IV (**Figure 2A**). However, the three *HbVIN* genes exhibited variation during the three developing leaf stages (I to III), with a constant rise for *HbVIN2* but different fluctuations for *HbVIN1* and *3*. Except for *HbNIN8* that accumulated transcripts throughout the leaf development, the other three *HbNIN* genes analyzed, namely *HbNIN1, 2* and *6* showed consistently higher expressions in developing leaves (stages I to III) than in mature leaves (stage IV) (**Figure 4B**), consistent with the higher NIN activity observed in developing leaves (**Figure 2B**). All three *HbCWI* genes displayed high expressions at stage III, but expression decreased significantly at stage IV in *HbCWI1* and *3* (**Figure 4C**); this trend was consistent with the changes in CWI activity (**Figure 2C**). The four *HbSus* genes displayed different patterns of expression during the four developmental leaf stages (**Figure 4D**). Expressions of both *HbSus2* and 3 increased constantly from stages I to III. At stage IV, *HbSus3* decreased abruptly to a barely detectable level whereas *HbSus2* increased further. Expression of *HbSus4* was the highest at stage I, and maintained high levels through stages II and III, but decreased significantly to a very low level at stage IV. *HbSus5* expression was much higher at late stages (III and IV) than the early stages (I and II). Both *HbSPS2* and *3* exhibited high gene activity in mature leaves at stage IV (**Figure 4E**), despite the lowest SPS enzyme activity observed at this stage (**Figure 2F**). Correlation analyses revealed that among the 16 sucrose-metabolizing related genes explored, expressions of only three genes, *viz. HbNIN2, HbCWI1* and *HbSus3*, correlated significantly with the activities of their respective enzymes (**Table 3**).

**Table 3:** Correlation coefficients between enzyme activity and gene expression in the course of *Hevea* leaf development.

Gene expression	Enzyme activity	Correlation coefficient
*HbVIN1*	VIN	-0.071
*HbVIN2*		0.407
*HbVIN3*		0.941
*HbNIN1*	NIN	0.884
*HbNIN2*		0.986^a^
*HbNIN6*		0.799
*HbNIN8*		-0.810
*HbCWI1*	CWI	0.999^b^
*HbCWI2*		0.711
*HbCWI3*		0.601
*HbSus2*	SSC	-0.245
*HbSus3*		0.957^a^
*HbSus4*		0.546
*HbSus5*		0.091
*HbSus2*	SSS	-0.244
*HbSus3*		0.959^a^
*HbSus4*		0.539
*HbSus5*		0.085
*HbSPS2*	SPS	-0.359
*HbSPS3*		-0.658

*^a^Significant at *P <* 0.05; ^b^Significant at *P <* 0.01. Correlation analysis was conducted using SPSS Statistics 22.*

## Discussion

Variation in the contents of different sugars during *Hevea* leaf development is consistent with the strong requirement of reducing sugars for energy and carbon skeletons in developing leaves (representing a sink), and the functions of mature leaves (source) that export excess photoassimilates mainly in the form of sucrose in higher plants (Turgeon, 1989). *Hevea* leaf area increased almost exponentially in the three developing stages, and peaked at stage III (pale-green) (**Table 1**). Similarly, hexose content of *Hevea* leaves increased with the leaf development, and also reached the maximum at stage III, being 1.57 times that of sucrose content (**Figure 1**). Such a leaf development requires a massive influx of water into the vacuoles (Pantin et al., 2012), and the high hexose contents observed in expanding *Hevea* leaves would facilitate the entry of water to establish high cell turgor pressure for rapid leaf expansion. A similar observation has been reported in immature grapevine leaves where the hexose content that represented more than 75% of the total carbohydrates decreased almost three fold in mature leaves (Patakas, 2000). High hexose contents are beneficial to the developing leaf as they are essential for fast cell division and expansion (da Silva Bonome et al., 2011). Moreover, the high hexose concentrations meet the requirement of a controlled cell-wall polymer synthesis that contributes to the extension of the pre-existing cell wall to accommodate cell growth (Cosgrove, 2005). High levels of hexose in developing leaves are due to the high activities of four types of sucrose-cleaving enzymes, i.e., VIN, NIN, CWI and Sus in the cleavage direction (SSC) (**Figure 2**). Similar results are obtained in peach, a sorbitol synthesizing species, where activities of VIN, NIN and SSC and the hexose content were the highest in the first rapidly expanding leaf (Merlo and Passera, 1991).

A common feature among sucrose-metabolizing enzymes in the course of *Hevea* leaf development is that these enzymes tended to be highly expressed in young leaves, with expression declining significantly at maturation, regardless of whether the enzymes were functioning in sucrose synthesis or cleavage (**Figure 2**). A similar trend has also been observed in eggplants, cassava, grapevine, sugar cane and maize (Claussen et al., 1985), lemongrass (Singh and Luthra, 1988), chicory (Gupta et al., 1991), common oak (Alaoui-Sossé et al., 1996), and sugar beet (Pavlinova et al., 2002). The synchronized change of sucrose cleavage and synthesis activity facilitates the keeping of sucrose levels within an appropriate range in *Hevea* leaves, an essential function critical for normal plant development (Yadav et al., 2014). In plant leaves, SPS activity is usually the rate-limiting enzyme for sucrose synthesis, whereas Sus activity is mainly involved in sucrose catabolism (Winter and Huber, 2000). At different developmental stages of *Hevea* leaves, however, Sus activity in the synthesis direction (SSS) was comparable or even higher than SPS (**Figure 2**). Of the four *HbSus* genes examined, expressions of *HbSus3* exhibited a significant positive correlation with both SSS and SSC enzyme activities (**Table 3**). On the other hand, expressions of neither *HbSPS* gene explored were consistent with the observed enzyme activity (**Figures 2F, 4E** and **Table 3**). This phenomenon where gene activity is not reflected in enzyme activity has also been observed in other plant species. For example, in ‘La France’ pear leaves (Suzue et al., 2006), SPS activity remained almost constant throughout the developmental stages although SPS mRNA levels are much higher in mature leaves than in young ones. Post-translational modification of the enzyme may play an essential role in controlling leaf SPS activity (Doehlert and Huber, 1983; Walker and Huber, 1989; Zhou et al., 2002).

One apparent discrepancy between *Hevea* and other plant species lies in the activities of NIN. In other plants reported so far, VIN is always the primary enzyme responsible for sucrose catabolism in developing or mature leaves, whereas NIN activities are much lower or even undetectable in leaves (Singh and Luthra, 1988; Huber, 1989; Alaoui-Sossé et al., 1996; Winter and Huber, 2000). For example, VIN activity was reported to be much higher in young leaves than in mature ones in many other plant species, including lemongrass (Singh and Luthra, 1988), common oak (Alaoui-Sossé et al., 1996), sugar beet (Pavlinova et al., 2002), and pear (Suzue et al., 2006). In this study, however, NIN exhibited activities comparable to the other three sucrose-cleaving enzymes (VIN, CWI and SSC) throughout all leaf stages (**Figure 2**), suggesting an active participation of NIN in *Hevea* leaf development. Of the four sucrose-cleaving enzymes, only NIN exhibited a negative correlation at a significant level for its expression with sucrose concentration in developing *Hevea* leaves, further corroborating its roles in this process (**Table 2**). Of the ten NIN genes identified in *Hevea* (Liu et al., 2015), eight displayed substantial expression in developing and/or mature leaves (**Figure 3**), providing additional evidence for the active roles of NIN in leaf development in the rubber tree.

Starch and sucrose are the principal photoassimilates accumulated in leaves of higher plants. However, the relative abundance of starch versus sucrose varies greatly among species. Among ten temperate grass species explored by Huber (1989), most had higher leaf starch content, and the ratios of starch to sucrose varied from about 22.0 in a soybean cultivar to about 0.5 in a pea cultivar. Similarly, of thirteen C_4_ species investigated (Lunn and Hatch, 1995), only two accumulated sucrose comparable to starch in their leaves during the day, whereas the others accumulated much more starch than sucrose. In mature leaves of young common oak, starch content was also much higher than sucrose (Alaoui-Sossé et al., 1996). Results from the present study showed that mature *Hevea* leaves had a sucrose:starch ratio of ∼11.0 (**Figure 1**), the highest value as compared with other plants reported to date. The leaves of cassava, another economically important tropical species within the same Euphorbiaceae family as Hevea, had a sucrose:starch ratio of ∼5.0 (Zhang et al., 2015). Of the grass species, timothy, an important cool-season herbage also showed a high sucrose:starch ratio of ∼6.0 in its leaves at stages of both heading and anthesis (Ould-Ahmed et al., 2017). However, this grass species is known to preserve fructans as the major non-structural carbohydrates in its leaves (Bélanger and McQueen, 1998). It is worth noting that leaf contents of both sucrose and starch exhibited a diurnal change in Arabidopsis (Smith et al., 2004), and the time of harvesting, therefore, might influence the sucrose:starch ratio in *Hevea* leaves, which were harvested this study at 10:00 am. *Hevea* leaves contained also small amounts of quebrachitol (Supplementary Figure S3), a cyclic polyol with medicinal uses reported in a small number of plant species (Díaz et al., 2008). Considering the high abundance [(about 1.2% (w/v)] of quebarchitol in *Hevea* latex (Bealing, 1969) and the presence of laticifers in the leaves, latex might be the source of the quebrachitol detected in *Hevea* leaves. The physiological functions of quebrachitol are still unclear in *Hevea* although a quebrachitol transporter (HbPLT2) has been identified in this species, and its expression was induced by ethylene treatment or wounding of the bark in a study (Dusotoit-Coucaud et al., 2010).

To conclude, this study provides the first atlas of developmental dynamics in activity and gene expression of sucrose-metabolizing enzymes as well as contents of various sugars in the leaves of *Hevea*, a tropical tree species. Although the behavior of certain genes and enzymes connected with sucrose metabolism mirrors that of other plants species, several novel characters show up. Unlike in most other species where VIN is the major sucrose-cleaving enzyme in developing leaves, NIN and Sus in the cleavage direction are no less prominent in *Hevea*. Whereas SPS is mainly responsible for sucrose synthesis in the leaves of most plant species, Sus in the synthesis direction is comparable or even higher than that of SPS in the different developmental stages of *Hevea* leaves. The findings obtained here would help in the understanding of the regulation of sucrose metabolism during leaf development in the plant kingdom and in the rubber tree in particular.

## Author Contributions

JZ conducted most of the experiments and data analysis, and drafted the primary manuscript. JQ coordinated the project. JHL helped develop the HPLC-ESLD method. XX and YF participated in gene expression analysis. JXL contributed in pilot studies. CT conceived the study, designed the experiments, and modified the manuscript. All the authors read and approved the manuscript.

## Conflict of Interest Statement

The authors declare that the research was conducted in the absence of any commercial or financial relationships that could be construed as a potential conflict of interest. The reviewer HCJvR and handling Editor declared their shared affiliation.
