# Supplementary material for: Characterization of Sugar Contents and Sucrose Metabolizing Enzymes in Developing Leaves of Hevea brasiliensis
Source: Front Plant Sci. 2018 Feb 1;9:58. doi: 10.3389/fpls.2018.00058 (PMC5799706; doi:10.3389/fpls.2018.00058)
Supplement: Supplementary file 1 [file Table_1.docx]

# Supplementary Tables

**Table S1.** Effect of UDP or UDPG on the blanks in assaying activities of sucrose synthase (Sus) and sucrose phosphate synthase (SPS) in *Hevea* leaves

| Enzyme | Protocol | OD_520_ readings for different leaf stage samples | | | |
| --- | --- | --- | --- | --- | --- |
|  |  | I | II | III | IV |
| SSC | With UDP | 0.60±0.01 | 0.59±0.00 | 0.59±0.00 | 0.60±0.01 |
|  | Without UDP | 0.60±0.01 | 0.59±0.01 | 0.60±0.02 | 0.60±0.00 |
| SSS | With UDPG | 0.14±0.00 | 0.13±0.01 | 0.13±0.00 | 0.15±0.00 |
|  | Without UDPG | 0.13±0.01 | 0.12±0.01 | 0.12±0.01 | 0.14±0.01 |
| SPS | With UDPG | 0.12±0.00 | 0.11±0.01 | 0.12±0.00 | 0.13±0.01 |
|  | Without UDPG | 0.11±0.01 | 0.11±0.01 | 0.10±0.01 | 0.12±0.00 |

**Table S2.** Specific PCR primers for main sucrose-metabolizing genes active over the course of *Hevea* leaf development.

| Gene | Forward primer(5'→3') | Reverse primer(5'→3') | |  |
| --- | --- | --- | --- | --- |
| *HbNIN1* | AAGAGGCAATCATGCAAACAA | | TGATAAAGGGCAAAGAATGGTG | |
| *HbNIN2* | GAAGAGAGGCAAACAAACAAG | | CGAAACCAAACATTTCTCACTTTAC | |
| *HbNIN6* | GTAGAATGGGTGTTTATGGGTATCC | | TCTACGAACTCTTTCCCCTCTT | |
| *HbNIN8* | AGTGCCAAAGGGCTGATAGTG | | ATGGTAATGTAGCTTCAGCAGCA | |
| *HbVIN1* | TGGTTTTCTTGTTTCTCGCTTT | | TGGCTTTCTCAGACACACCG | |
| *HbVIN2* | TCTTGTTGATGACAGCCTTGC | | TGATTGTCGTTCTTCCCCCTT | |
| *HbVIN3* | CAAACTAGCCTTTAGCACA | | TATCAAACCACCAACTTCA | |
| *HbCWI1* | ATAGGAGGATATTATGGGGCTG | | ATGCTGGATGCTGACTTGCT | |
| *HbCWI2* | AACCTCCATCTTCCATCAACAC | | ACCCATCTGACCCGTACCTC | |
| *HbCWI3* | CTAAACAACGCCAAATCCAAA | | TCCCTATCAAGGTCCTCCAAT | |
| *HbSus2* | CGGACGAGTGGATAGGTAAA | | AACCAGCAATCACAACAAGG | |
| *HbSus3* | TGAAAGAGTCGGGCGAGGGA | | TCGTTGTTGAGGAGTTGCGTGTT | |
| *HbSus4* | TGTTGTTCATGGTATTGATGTCTTT | | CACGCACAAGTGTTCTTCATT | |
| *HbSus5* | TATGTCCATCTACTTCCCTTACTCT | | GATGTATCCAGCAACCACGAC | |
| *HbSPS2* | CTGCCTCAATCAAAGTAAGCCCGAA | | ATGGGTAACCGAGATGGAAT | |
| *HbSPS3* | TCATCCGACCAAACTTCC | | GGGACAACAAAGACTGGC | |
| *YLS8* | CCTCGTCGTCATCCGATTC | | CAGGCACCTCAGTGATGTC | |

**Supplementary Figures**

**Figure S1**


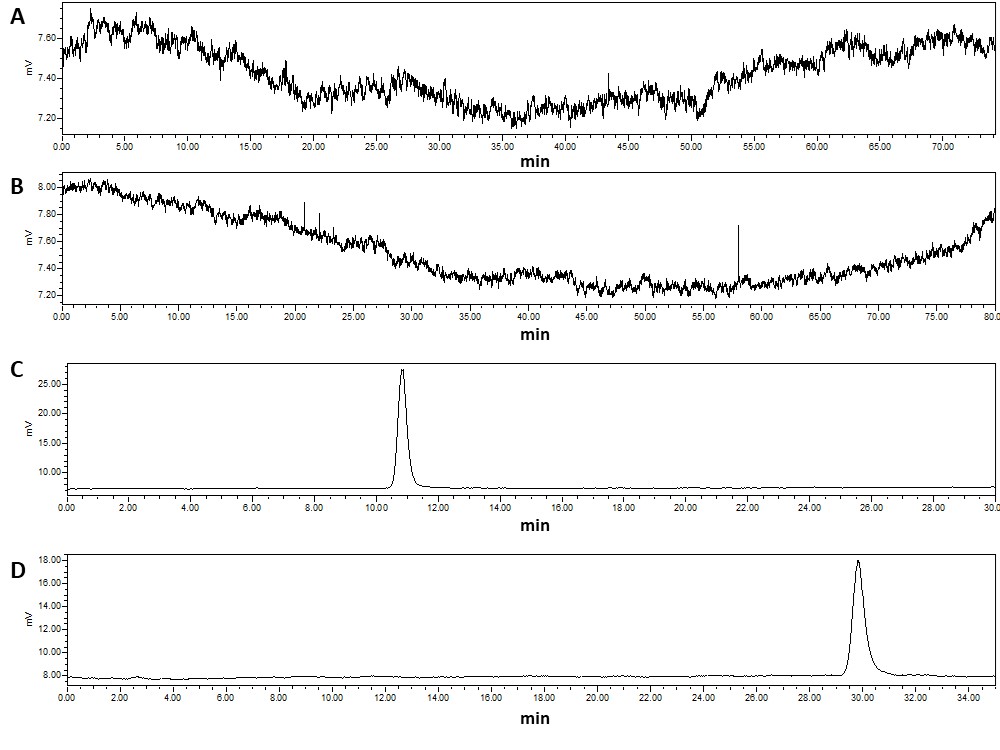


**Supplemental Figure 1.** UDP (A) and UDPG (B) at a concentration of 1 mg/mL were analysed by HPLC-ESLD together with the standard sugars fructose (C) and sucrose (D). This methodology fails to detect UDPG and UDP, but it is very sensitive for the detection of glucose, fructose and sucrose (detection limit 5 µg/mL). We refer to the legend of Fig. S2B for more details on the separation.

**Figure S2**

**
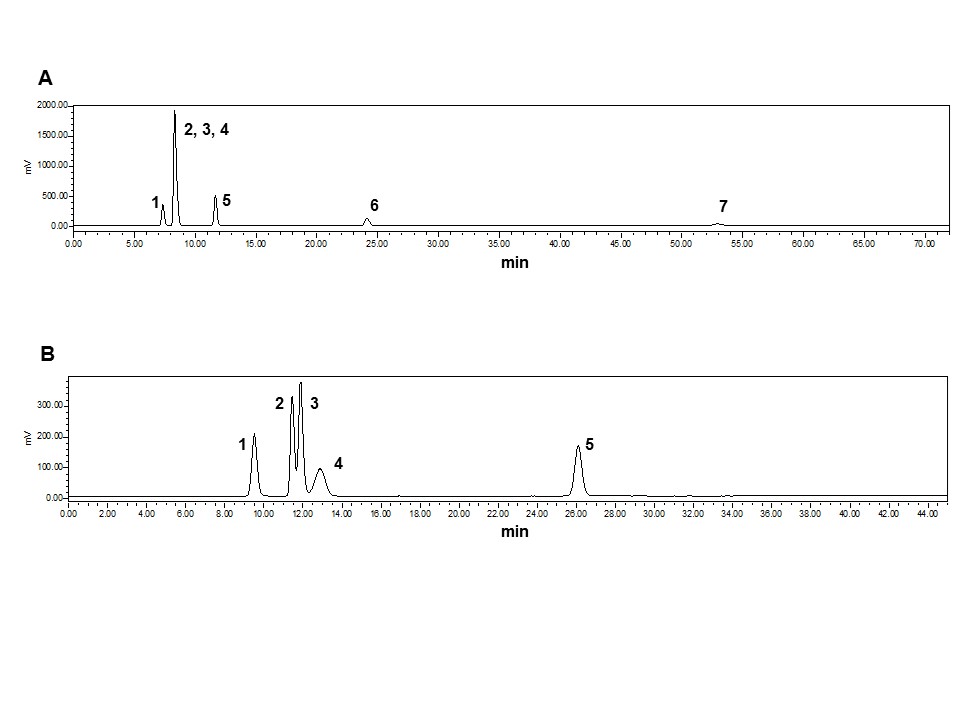
**

**Supplemental Figure 2.** HPLC–ELSD chromatogram of standard sugar peaks. A mixture of 10 μL 1mg/mL each of standard substance was injected on XBridge^TM^ Amide column (4.6 mm × 250 mm i.d., 3.5 μm particle size). **(A)** The separation was conducted using the solvent of acetonitrile:water 75: 15 (v/v) at a flow rate of 1 mL/min, a drift tube temperature of 82 ^o^C, a column temperature of 30 ^o^C and a nebulizer gas flow rate of 2 L/min. **(B)** The separation was conducted using the solvent of acetonitrile:water 85: 15 (v/v) at a flow rate of 1 mL/min, a drift tube temperature of 82 ^o^C, a column temperature of 45 ^o^C and a nebulizer gas flow rate of 2 L/min. Sugar peaks are fructose (1), quebrachitol (2), sorbitol (3), glucose (4), sucrose (5), raffinose (6), and stachyose (7).

**Figure S3**

**
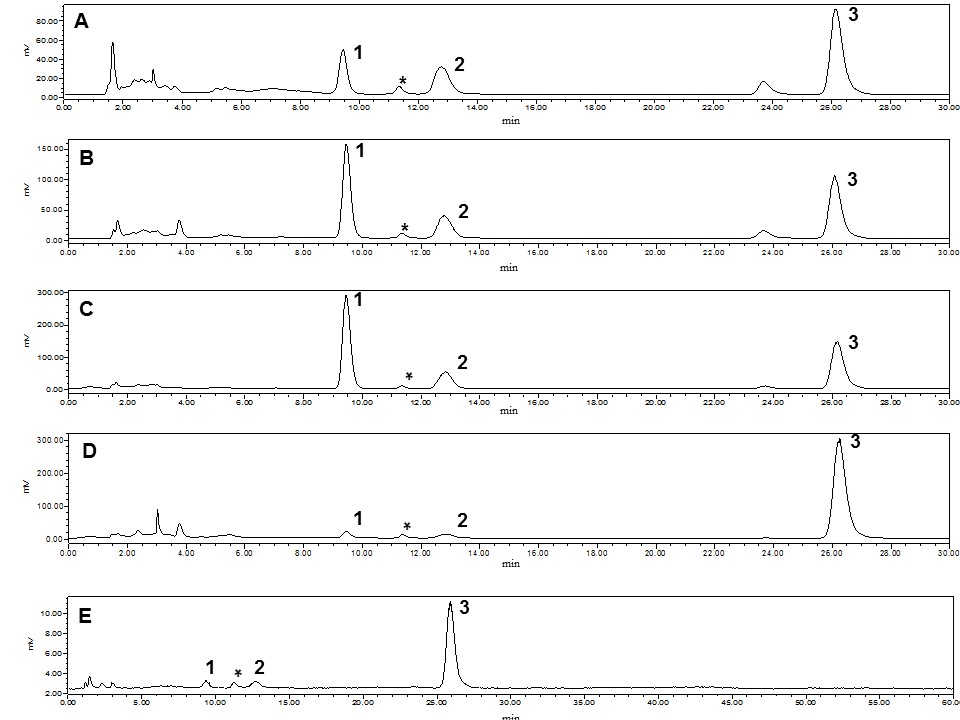
**

**Supplemental Figure 3.** HPLC–ELSD chromatogram of soluble sugars in *Hevea* leaves at stages I **(A)**, II **(B)**, III **(C)**, and IV **(D)**, and phloem exudates **(E)**. Peaks 1, 2 and 3 represent fructose, glucose and sucrose, respectively. The peak indicated by an asterisk represents quebrachitol. The separation was conducted using the solvent of acetonitrile:water 85: 15 (v/v) at a flow rate of 1 mL/min, a drift tube temperature of 82 ^o^C, a column temperature of 45 ^o^C and a nebulizer gas flow rate of 2 L/min.
